# Generation of broadband ultraviolet frequency-entangled photons using cavity quantum plasmonics

**DOI:** 10.1038/s41598-017-08431-x

**Published:** 2017-08-14

**Authors:** Hisaki Oka

**Affiliations:** Institute for Research Promotion, Niigata University, 8050, Ikarashi 2-no-cho, Nishi-ku, Niigata 950-2181 Japan

## Abstract

Application of quantum entangled photons is now extending to various fields in physics, chemistry and biology. In particular, in terms of application to molecular science, broadband ultraviolet frequency-entangled photons are desired because molecules inducing photochemical reactions of interest often have electronic transition energies in the ultraviolet region. Recent standard method for generating such entangled photons is a chirped quasi-phase-matching method, however this method is not suitable for the generation of ultraviolet frequency-entangled photons because it requires down-conversion of a photon with a wavelength shorter than ultraviolet into an entangled photon pair. Here we propose a simple method for generating broadband ultraviolet frequency-entangled photons using cavity quantum plasmonics, in which conventional cavity quantum electrodynamics theory is applied to quantum plasmonics. We introduce a cavity-plasmon system in which localised surface plasmon (LSP) is coupled to the cavity fields of a state-of-the-art microcavity. Using this system, we theoretically show that broadband ultraviolet frequency-entangled photons can be generated simply by utilising the absorption saturation effect of LSP.

## Introduction

Quantum entangled photons have been investigated primarily in optical quantum information technologies; however the research target is now extending to various fields of application, such as quantum measurement, quantum spectroscopy and coherent control of molecules^1–4^. In applications to such research fields, broadband frequency entanglement, control of quantum correlation and high fluxional generation are required rather than conventional polarisation entanglement and on-demand generation of entangled photons. In fact, in molecular two-photon processes, enhancement and control of population of a vibrational state by frequency-entangled photons have been reported^5, 6^. In addition, in the field of spectroscopy, a measurement method for virtual states in molecules using tailored quantum-correlated photons has been theoretically predicted^7, 8^. These phenomena are not induced by classical laser and therefore an entirely new optical control of molecules is expected.

At present, the standard method for generating broadband frequency-entangled photons is to use a chirped quasi-phase-matching method^9^ in which the target is restricted almost exclusively to the visible and communication wavelength bands^10–12^. In terms of application to molecular science, however, ultraviolet frequency-entangled photons are desired because molecules inducing photochemical reactions of interest often have electronic transition energies in the ultraviolet region. In general, the chirped quasi-phase-matching method is not suitable for the generation of ultraviolet frequency-entangled photons because it requires down-conversion of a photon with a wavelength shorter than ultraviolet into an entangled photon pair.

One powerful candidate for generating such ultraviolet frequency-entangled photons is a method utilising localised surface plasmon (LSP)^13^. LSP is quantised plasma oscillation formed near the surface of a nanometal and is induced by directly irradiating light with wavelengths in the ultraviolet region, namely, around 350 nm for silver^14, 15^ and around 200 nm for aluminium^14, 16–18^. In addition, LSP can focus light to the nanometre scale and strongly enhances electric field near nanometals, so-called antenna effect. In fact, many studies utilising the antenna effect, such as enhancements of molecular fluorescence^19, 20^ and of two-photon absorption^21^, entanglement generation between qubits^22^ and application to single photon sources^23, 24^, have been reported. Furthermore, it has been experimentally observed that LSP exhibits a rapid radiative decay time shorter than 10 fs, corresponding to a spectral linewidth larger than ~300 meV^25^. Since rapid radiative decay directly leads to spectral broadening of photon emission, generation of broadband ultraviolet frequency-entangled photons can be expected by utilising the LSP as an emitter.

As is well known, however, the optical response of LSP is often analysed by classical electromagnetism^26^, in spite of the fact that LSP is quantised plasma oscillation. This is because that the electric-field enhancement of LSP can be explained simply by Maxwell’s equations. In fact, bio-sensing^27^ and molecular fluorescence enhancement^28^, which are major applications in the field of plasmonics, can be understood without the quantum nature of plasmon. However, in the analysis of entangled-photon generation, this quantum nature of plasmon is required and therefore Maxwell’s equations are unavailable. The research field dealing with the quantum nature of plasmon is called “quantum plasmonics^29^”, and has attracted much attention over the past few years. However, quantum plasmonics require quantum electrodynamics (QED) theory based on the second quantisation of plasmon and the derivation is often complicated whereas the description is rigorous. This might be a bottleneck restricting the spread of this research field.

Here, we introduce a simple way to quantise LSP and propose a simple method for generating broadband ultraviolet frequency-entangled photons using cavity quantum plasmonics. In what follows, we explain our strategy step by step. First, we introduce a simple way to quantise LSP by using an effective dipole approximation. Though analytical rigour becomes slightly deteriorated, the analysis of LSP can be reduced to an effective quantum two-level system and is therefore significantly simplified in comparison with conventional rigorous approaches. Next, we apply the framework of cavity-QED theory to our simplified quantum plasmonics (which we call cavity quantum plasmonics) and introduce a controlled cavity-plasmon system in which LSP can act as a photon emitter with directional emission. We show that this can be achieved by confining a single nanometal within a state-of-the-art microcavity whose cavity parameters are carefully chosen. Finally, we propose a simple method to generate broadband ultraviolet frequency-entangled photons by using the cavity-plasmon system. For sliver and aluminium nanometals as examples, we theoretically show that broadband ultraviolet frequency entangled photons can be generated simply by utilising the absorption saturation effect of LSP. In our cavity-plasmon system, cavity QED parameters are carefully chosen such that LSP can coherently interact with incident photons through cavity modes and be saturated with one-photon absorption. Therefore, either one of two input photons is absorbed by the LSP and the other remains unabsorbed owing to the absorption saturation effect of LSP. Photon entanglement is formed as a consequence of the interference between the unabsorbed photon and photon re-emitted from the LSP.

## Results

### Second quantisation of the localised surface plasmon: A simple method using effective dipole approximation

**Figure 1:**
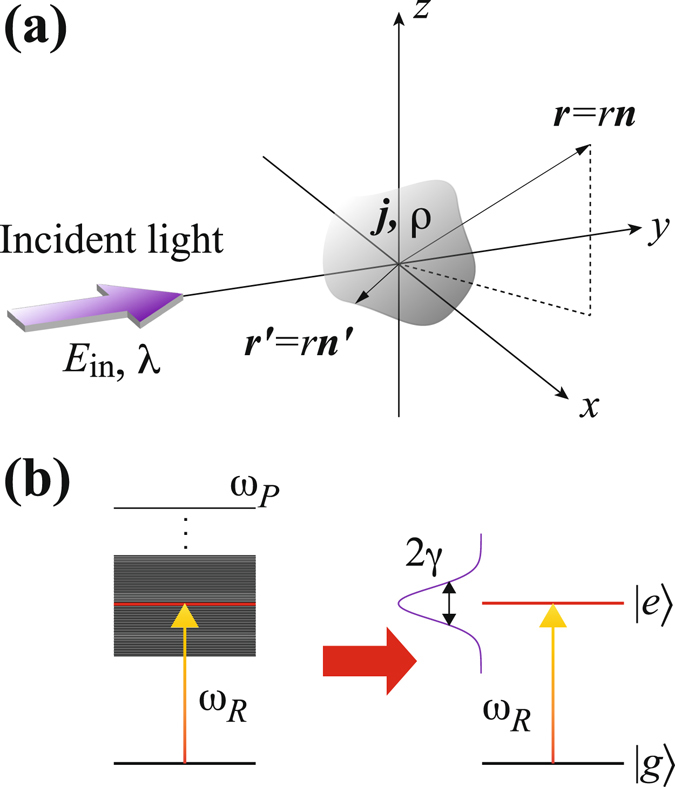
(**a**) Schematic of the analytical model. A nanometal is located at the origin and interacts with incident light ${{\boldsymbol{E}}}_{{\rm{i}}n}$. ${{\boldsymbol{r}}}^{^{\prime} }$ is defined within the nanometal and ***r*** is defined outside it ($r\ge r\text{'}$). ***n*** and ***n***′ are the unit vectors of ***r*** and ***r***′, respectively. (**b**) Effective quantum two-level system of LSP, where ${\omega }_{P}$ is the plasma frequency and the red line indicates the plasmon resonance.

We start by considering the interaction between an arbitrarily shaped nanometal with a finite volume and incident light ${{\boldsymbol{E}}}_{{\rm{in}}}$ with wavelength λ. The corresponding analytical model is depicted in Fig. 1(a). The size of the nanometal is characterised by ${r}^{\prime}$. ${{\boldsymbol{E}}}_{{\rm{in}}}$ induces the internal current density ***j*** and polarised charge density *ρ* within the nanometal, and the vector potential ***A*** and electrostatic potential *ϕ* are generated by ***j*** and *ρ*, respectively. Assuming $|r\text{'}|\equiv r\text{'}\ll \lambda$, ${\boldsymbol{A}}({\bf{r}},t)$ and $\varphi (r,t)$ outside the nanometal ($r > r\text{'}$) can be approximated as

1 $${\boldsymbol{A}}({r},t)\approx \frac{{\mu }_{0}}{4\pi }\sum _{\ell \mathrm{=1}}\frac{\mathrm{(2}\ell -\mathrm{1)(}-r{)}^{\ell -1}}{\mathrm{(2}\ell -\mathrm{1)!!}}{(\frac{d}{rdr})}^{\ell -1}(\frac{1}{r}\langle {{\bf{j}}}^{(\ell -\mathrm{1)}}(t)\rangle ),$$

2 $$\varphi (r,t)\approx \frac{1}{4\pi \varepsilon }\sum _{\ell =0}\frac{(2\ell +1)(-r{)}^{\ell }}{(2\ell +1)!!}{(\frac{d}{rdr})}^{\ell }(\frac{1}{r}\langle {\rho }^{(\ell )}(t)\rangle),$$

where we introduce the notation $\langle {f}^{(\ell )}(t)\rangle=\int d{\boldsymbol{r}}\text{'}r{\text{'}}^{\ell }{P}_{\ell }(\cos \,\alpha )f({\boldsymbol{r}}\text{'},t-r/c)$. ${P}_{\ell }$ is the Legendre polynomial and $\cos \,\alpha ={\boldsymbol{r}}\cdot {\boldsymbol{r}}\text{'}/rr\text{'}$. For $\ell \,=\,0$, *ϕ* is simply a static electric field and is ignored in this study.

We restrict ourselves to dipole radiation, for simplicity, because quadrupole and magnetic-dipole radiations are negligibly small. Limiting $\ell$ to $\ell =1$ in equations (1) and (2), the electric field near the nanometal is obtained from the relation ${\boldsymbol{E}}=-\partial {\boldsymbol{A}}/\partial t-\nabla \varphi$ and is described as

3 $${\boldsymbol{E}}({\boldsymbol{r}},t)=\frac{1}{4\pi \varepsilon }\frac{3{\boldsymbol{n}}[{\boldsymbol{n}}\cdot {\boldsymbol{p}}(t-r/c)]-{\boldsymbol{p}}(t-r/c)}{{r}^{3}},$$

where ***p*** is the effective dipole moment, defined as

4 $${\boldsymbol{p}}(t)=\int d{\boldsymbol{r}}\text{'}\,{\boldsymbol{r}}\text{'}\rho ({\boldsymbol{r}}\text{'},t\mathrm{).}$$

Consequently, under the condition of $r\text{'}\ll \lambda$, the localised surface plasmon (LSP) for a small arbitrary-shaped nanometal can be described by equation (3) and the influence of the shape of the nanometal can be reduced to the effective dipole moment, Eq. (4). For large nanometals (${\boldsymbol{r}}\text{'}\ge \lambda$), though not considered in this study, we only have to take into account $\ell \ge 2$ and use the Mie scattering theory.

The second quantisation of LSP is performed through ***p***. When we focus on the plasmon excitation at low-intensity light, ***p*** can be described by the linear response of ${{\boldsymbol{E}}}_{{\rm{in}}}$ as^26^

5 $${\boldsymbol{p}}={\varepsilon }_{0}{\varepsilon }_{m}\alpha {{\boldsymbol{E}}}_{{\rm{in}}},$$

where ${\varepsilon }_{m}$ is the dielectric constant of the background and *α* is the polarisability that depends on the complex dielectric function $\varepsilon (\omega )$ of the nanometal. Our strategy is then to reduce ***p*** to the quantum-mechanical one, ***p***_*Q*_, obtained from a two-level system with transition energy ${\omega }_{R}$,

6 $${{\boldsymbol{p}}}_{Q}=\frac{{|{\boldsymbol{d}}|}^{2}}{\hslash }\frac{1}{{\omega }_{R}-\omega -i\gamma }{{\boldsymbol{E}}}_{{\rm{in}}},$$

where ***d*** is the dipole moment and *γ* is the dipole relaxation rate. For the incident light with a frequency close to the plasmon resonance frequency ${\omega }_{R}$, *α* in equation (5) can be approximated by taking the Taylor expansion of the complex dielectric function $\varepsilon (\omega )$ around ${\omega }_{R}$. For spherical and ellipsoidal nanometals, the explicit expressions of *α* are already given^26^. In what follows, we take a spherical nanometal with a radius of *α*, for simplicity. *α* for spherical metal is given by

7 $$\alpha =4\pi {a}^{3}\frac{\varepsilon (\omega )-{\varepsilon }_{m}}{\varepsilon (\omega )+2{\varepsilon }_{m}}\mathrm{.}$$

The plasmon resonance enhancement takes place under the condition of ${\rm{Re}}[\varepsilon ({\omega }_{R})]=-2{\varepsilon }_{m}$. In the case of small or slowly varying ${\rm{Im}}[\varepsilon (\omega )]$ around ${\omega }_{R}$, $\varepsilon (\omega )$ can be approximated by taking the Taylor expansion, given by

8 $$\varepsilon (\omega )\approx -2{\varepsilon }_{m}+\frac{d{\rm{Re}}[\varepsilon ({\omega }_{R})]}{d\omega }(\omega -{\omega }_{R})+i{\rm{Im}}[{\omega }_{R}],$$

where we use ${\rm{Re}}[\varepsilon ({\omega }_{R})]=-2{\varepsilon }_{m}$ and $d{\rm{Im}}[\varepsilon ({\omega }_{R})]/d\omega \approx 0$. By substituting equation (8) into equation (7), we obtain

9 $$\alpha \approx 4\pi {a}^{3}(1+\frac{3{\varepsilon }_{m}}{\eta }\frac{1}{{\omega }_{R}-\omega -i\gamma }),$$

where $\eta =d{\rm{Re}}[\varepsilon ({\omega }_{R})]/d\omega$ and $\gamma ={\rm{Im}}[\varepsilon ({\omega }_{R})]{\eta }^{-1}$. The factor of $3{\varepsilon }_{m}{\eta }^{-1}$ yields the enhancement of the electric field by plasmon resonance. For the condition of large plasmon enhancement, $|3{\varepsilon }_{m}{\eta }^{-1}|\gg 1$, the first term in parentheses can be ignored and ***p*** can read

10 $${\boldsymbol{p}}=\frac{12{\varepsilon }_{0}{\varepsilon }_{m}^{2}\pi {a}^{3}}{\eta }\frac{1}{{\omega }_{R}-\omega -i\gamma }{{\boldsymbol{E}}}_{{\rm{in}}}\mathrm{.}$$

By assuming $12{\varepsilon }_{0}{\varepsilon }_{m}^{2}\pi {a}^{3}{\eta }^{-1}={|{\boldsymbol{d}}|}^{2}{\hslash }^{-1}$, equation (10) becomes the same as equation (6). The second quantisation can be performed simply by replacing ***p*** with $\hat{{\boldsymbol{p}}}={\boldsymbol{d}}({\hat{\sigma }}^{+}+{\hat{\sigma }}^{-})$ by introducing the effective dipole moment ***d*** and the dipole operators ${\hat{\sigma }}^{-}=|g\rangle \langle e|$ and ${\hat{\sigma }}^{+}=|e\rangle \langle g|$, where $|g\rangle$ and $|e\rangle$ are the ground and excited states of the LSP, respectively. The difference in shape of nanometal is included in ***d***. The Hamiltonian of the LSP can then be simply described as ${\hat{H}}_{{\rm{LSP}}}=\hslash {\omega }_{R}{\hat{\sigma }}^{+}{\hat{\sigma }}^{-}$. Thus, the LSP can be reduced to an effective quantum two-level system with plasmon resonance frequency ${\omega }_{R}$ and dipole relaxation rate *γ*, as depicted in Fig. 1(b).

The effective dipole moment ***d*** can be derived from the relation, ${|{\boldsymbol{d}}|}^{2}{\hslash }^{-1}=12{\varepsilon }_{0}{\varepsilon }_{m}^{2}\pi {a}^{3}{\eta }^{-1}$, e.g. by using the Drude model of $\varepsilon (\omega )={\varepsilon }_{\infty }-{\omega }_{P}^{2}{({\omega }^{2}+i2\omega \gamma )}^{-1}$, where ${\omega }_{P}$ is the plasma frequency and ${\varepsilon }_{\infty }$ is the dielectric constant of nanometal. Using $\eta =d{\rm{R}}e[\varepsilon ({\omega }_{R})]/d\omega$ and presuming ${\omega }_{P}^{2}\gg {\gamma }^{2}$, we can simply derive the dipole moment $|{\boldsymbol{d}}|$ as

11 $$|{\boldsymbol{d}}|=\frac{{\mathrm{(6}{\varepsilon }_{0}{\varepsilon }_{m}^{2}\pi {a}^{3}\hslash {\omega }_{P})}^{\mathrm{1/2}}}{{({\varepsilon }_{\infty }+2{\varepsilon }_{m})}^{\mathrm{3/4}}}\mathrm{.}$$

The dipole relaxation rate *γ* can also be evaluated from the relation $2\gamma ={\omega }_{R}^{3}{|{\boldsymbol{d}}|}^{2}\mathrm{/3}\pi {\varepsilon }_{m}{\varepsilon }_{0}\hslash {c}^{3}$ by using equation (11). The method stated above can be simply extended to the LSP of an ellipsoidal nanometal. Similar and more exact derivations of plasmon second quantisation can be found in Refs 30 and 31.

### A nanometal coupled to a microcavity: One-dimensional cavity-plasmon system

In this section, we introduce a cavity-plasmon system in order to treat the LSP as an emitter with directional emission. In general, photons emitted from a nanometal are radiated in all directions. From the viewpoint of an entangled photon source, this is undesirable and therefore we have to restrict the direction of photon emission from the LSP. To achieve this, we utilise the cavity quantum electrodynamics (QED) effect.

**Figure 2:**
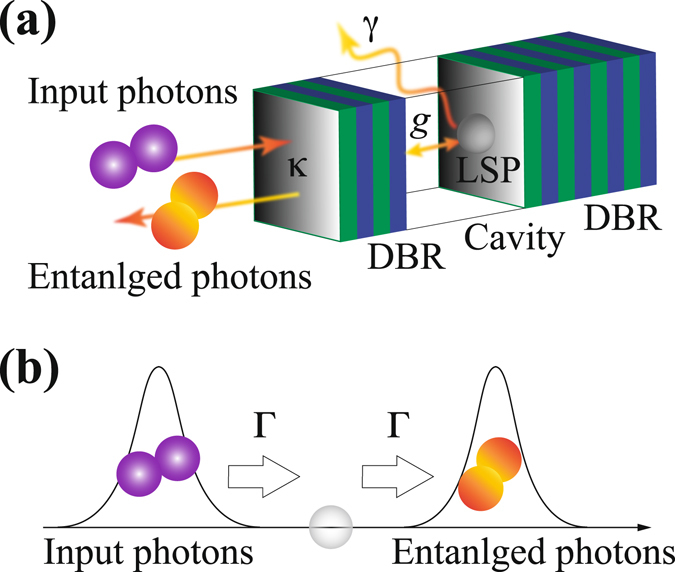
(**a**) Schematic of the conversion of input photons to an entangled photon pair via a cavity-plasmon system in which a single nanometal is confined in a microcavity. (**b**) One-dimensional input-output model of the cavity-plasmon system simplified under the condition of $\kappa  > g > \gamma$.

A schematic of the cavity-plasmon system is depicted in Fig. 2(a), where a nanometal is embedded in a microcavity system such as a distributed Bragg reflector (DBR) microcavity. *κ* and *g* are conventional cavity QED parameters, namely the cavity damping rate and LSP-cavity coupling rate, respectively. *γ* is the dipole relaxation rate of LSP, introduced in the previous section. We impose the condition $\kappa  > g > \gamma$ upon the cavity-plasmon system, which is called a one-dimensional input-output system^32, 33^. Under the first condition ($\kappa  > g$), photon leakage through the cavity field exceeds the cavity-plasmon interaction and therefore the cavity field can be eliminated by introducing an effective damping rate of the cavity-plasmon system, ${\rm{\Gamma }}={g}^{2}/\kappa$. In addition, the second condition ($g > \gamma$) ensures that incident photons can coherently interact with the LSP through the cavity field. Thus, the condition $\kappa  > g > \gamma$ is the intermediate regime between the weak- and strong-coupling regimes in conventional cavity QED and requires no high-$Q$ microcavity.

The role of the microcavity is only to realise a high spontaneous emission factor of $\beta \simeq 1$ ^34^. *β* is defined as the ratio of spontaneous emission through a cavity to the total spontaneous emission including that into free space (all directions), $\beta ={\rm{\Gamma }}/({\rm{\Gamma }}+\gamma )$. Therefore, *β* = 1 indicates that all photon emission from the cavity-plasmon system occurs through the cavity field (no loss into free space), and the analytical model can be simply reduced to a one-dimensional model^35^, as depicted in Fig. 2(b). Thus, assuming the condition $\kappa  > g > \gamma$ and a high *β* factor, we can treat the LSP as an emitter with rapid radiative decay and high directivity, which are useful for a broadband frequency-entangled photon source.

### Ultraviolet frequency-entangled photon generation

Finally, we propose a simple method to generate an ultraviolet frequency-entangled photon pair using the one-dimensional cavity-plasmon system. Our strategy is to utilise the absorption saturation effect of LSP confined in the microcavity. The total Hamiltonian $\hat{H}$ can now be described as

12 $$\begin{array}{c}\hat{H}={\omega }_{R}{\hat{\sigma }}^{+}{\hat{\sigma }}^{-}+\int d\omega \,\omega {\hat{a}}^{\dagger }(\omega )\hat{a}(\omega )\\ \quad \quad +\int d\omega \,\sqrt{\frac{{\rm{\Gamma }}}{\pi }}({\hat{a}}^{\dagger }(\omega ){\hat{\sigma }}^{-}+{\hat{\sigma }}^{+}\hat{a}(\omega )),\end{array}$$

where the natural unit of $\hslash =c=1$ is used. $\hat{a}(\omega )$ and ${\hat{a}}^{\dagger }(\omega )$ are the annihilation and creation operators of a photon with frequency $\omega$, respectively. For a cavity-plasmon system with high *β* factor ($\simeq 1$), radiative loss into free space is negligible. Therefore, in such a cavity-plasmon system, the dynamics of the total system can be calculated using the Schrödinger equation,

13 $$|{\rm{\Psi }}(t)\rangle =\exp (-i\hat{H}t)|{\rm{\Psi }}\mathrm{(0)}\rangle ,$$

where $|{\rm{\Psi }}\mathrm{(0)}\rangle$ is the initial state of the total system, defined as $|{\rm{\Psi }}(0)\rangle=|\psi {\rangle}_{{\rm{i}}{\rm{n}}}\otimes |g\rangle$. $|\psi {\rangle}_{{\rm{i}}{\rm{n}}}$ indicates the quantum state of incident photons.

In order to analyse the conversion of two photons into an entangled-photon pair, we consider an input pulse of exactly two photons as $|\psi {\rangle}_{{\rm{i}}{\rm{n}}}$, given by^36^

14 $$|\psi {\rangle}_{{\rm{i}}{\rm{n}}}\,{=2}^{-1/2}\int d\omega \int d\omega \text{'}{\psi }_{{\rm{i}}{\rm{n}}}(\omega ,\omega \text{'}){\hat{a}}^{\dagger }(\omega ){\hat{a}}^{\dagger }(\omega \text{'})|0\rangle,$$

where $\psi (\omega ,\omega \text{'})$ is the wave function of the two photons. For ${\psi }_{{\rm{i}}{\rm{n}}}(\omega ,\omega \text{'})$, we adopt an uncorrelated two-photon Gaussian pulse with a central frequency ${\omega }_{R}$ resonant with the plasmon resonance frequency and a pulse width *σ*,

15 $${\psi }_{{\rm{i}}{\rm{n}}}(\omega ,\omega \text{'})=\psi (\omega )\psi (\omega \text{'}){\rm{w}}{\rm{i}}{\rm{t}}{\rm{h}}\psi (\omega )\propto \exp [-{(\omega -{\omega }_{R})}^{2}{\sigma }^{2}/2].$$

This can be experimentally generated, e.g. by utilising the scheme reported in ref. 37. By the interaction with the cavity-plasmon system, ${\psi }_{{\rm{in}}}(\omega ,\omega \text{'})$ is converted into an entangled-photon pair, ${\psi }_{{\rm{out}}}(\omega ,\omega \text{'})\ne \psi (\omega )\psi (\omega \text{'})$.

In the actual calculation, we numerically solve equation (13) by discretising the continuous photon fields and evaluate the degree of entanglement of the output photons by employing the entropy of entanglement, *E* ^38^. We target aluminium and silver nanospheres because the Drude model is relatively applicable in the ultraviolet region. The concrete calculation method and the cavity-plasmon parameters are given in the section of Methods. In what follows, we clarify the optimal condition of pulse width *σ*_*m*_, yielding the maximum *E*, by changing *σ* of input photons.

**Figure 3:**
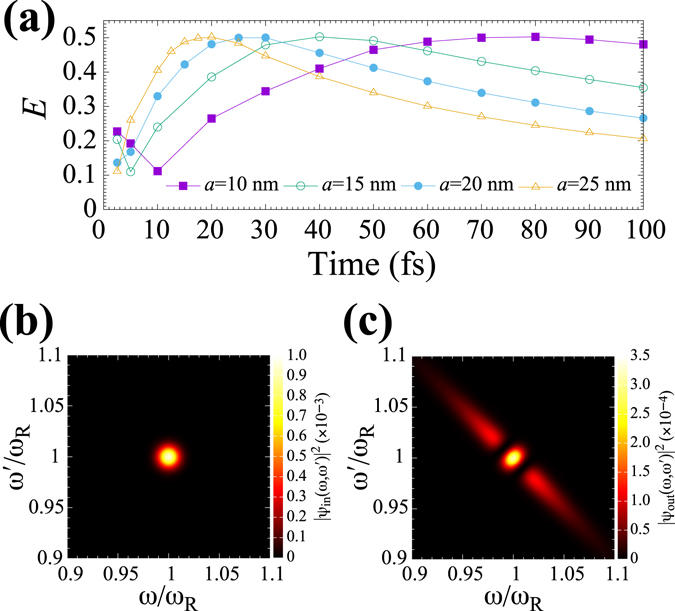
(**a**) *E* as a function of *σ* for silver nanospheres of *a* = 10, 15, 20 and 25 nm. (**b**) ${|{\psi }_{{\rm{in}}}(\omega ,\omega \text{'})|}^{2}$ with *σ* = 20 fs. (**c**) ${|{\psi }_{{\rm{out}}}(\omega ,\omega \text{'})|}^{2}$ yielding the maximal *E* for *a* = 25 nm. The solid lines in (**a**) are to guide the reader’s eye.

To start with, we analyse the entangled photon generation obtained from the cavity-plasmon system with silver nanospheres. In our calculation parameters, the plasmon resonance frequency is ${\omega }_{R}=3.222$ eV, corresponding to 385 nm. Figure 3(a) shows *E* as a function of *σ* for a = 10, 15, 20 and 25 nm. One can see that *E* reaches its peak at a specific pulse width *σ*_*m*_ for each *a* and that the value of *σ*_*m*_ becomes smaller for larger *a*: *σ*_*m*_ = 80 fs for *a* = 10 nm, *σ*_*m*_ = 40 fs for *a* = 15 nm, *σ*_*m*_  = 30 fs for *a*  = 20 nm and *σ*_*m*_  = 20 fs for *a*  = 25 nm. On the other hand, the maximum values of *E* at *σ*_*m*_ are little dependent upon the change in *a* and are constantly $E\approx 0.5$. The conversion of input photons to entangled photons can be visualised and simply understood by calculating the two-photon joint spectrum^39^, ${|\psi (\omega ,\omega \text{'})|}^{2}$, defined in the $\omega -\omega \text{'}$ plane. Figure 3(b) and (c) show ${|{\psi }_{{\rm{i}}{\rm{n}}}(\omega ,\omega \text{'})|}^{2}$ and ${|{\psi }_{{\rm{o}}{\rm{u}}{\rm{t}}}(\omega ,\omega \text{'})|}^{2}$, respectively, yielding the maximum *E* for *a* = 25 nm. One can find that the input photons with no correlation (isotropic distribution) are converted into photon distribution with negative frequency correlation. This anisotropic distribution with negative frequency correlation indicates frequency entanglement between photons and is characterised by two pulse widths: the pulse width along the short axis of $\omega =\omega \text{'}$ and that along the long axis of $\omega +\omega \text{'}=2{\omega }_{R}$. The former is characterised by ${\sigma }^{-1}$ and the latter is equal to $2{\rm{\Gamma }}$ = 306 meV for *a* = 25 nm. Though not shown in the figure, the two-photon joint spectra of the outputs obtained from other *σ*_*m*_ for *a* = 10, 15 and 20 nm are little different in shape from Fig. 3(c), except for the value of pulse width $2{\rm{\Gamma }}$ (see the Methods section for details). Thus, for silver nanospheres, frequency entangled photons with pulse width ~300 meV and central wavelength 385 nm can be obtained under the present parameters.

**Figure 4:**
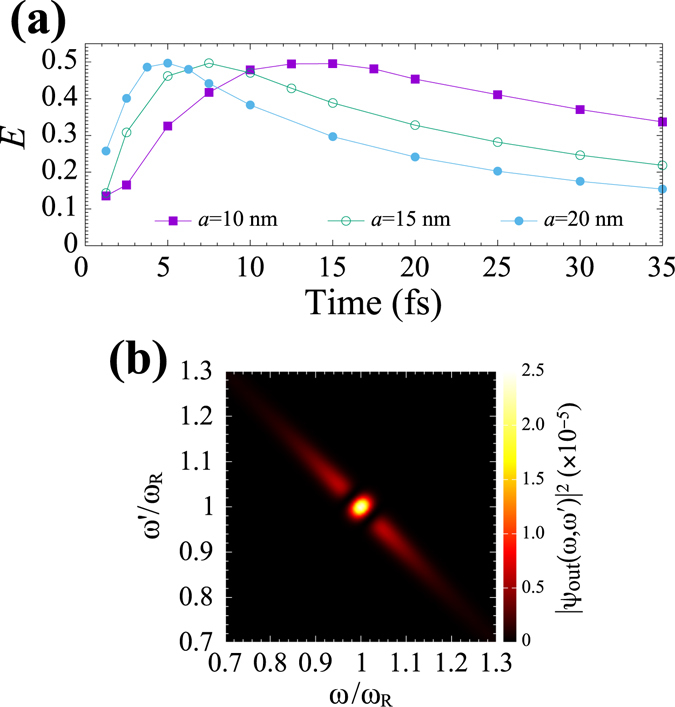
(**a**) *E* as a function of *σ* for aluminium nanospheres of *a* = 10, 15 and 20 nm. (**b**) ${|{\psi }_{{\rm{out}}}(\omega ,\omega \text{'})|}^{2}$ yielding the maximal *E* for *a* = 20 nm. The solid lines in (**a**) are to guide the reader’s eye.

Next, we analyse the entangled photon generation by the cavity-plasmon system with aluminium nanospheres. In our calculation parameters, the plasmon resonance frequency is ${\omega }_{R}=5.769$ eV, corresponding to 215 nm. Figure 4(a) shows *E* as a function of *σ* for *a* = 10, 15 and 20 nm. *E* exhibits almost the same tendency as that for silver nanospheres, except for the result that *σ*_*m*_ yielding the maximum *E* shifts to smaller values up to 5 fs, even for *a* = 20 nm. In addition, as is the case with silver nanospheres, the maximum values of *E* are little dependent upon the change in *a* and are constantly $E\approx 0.5$. Furthermore, as shown in Fig. 4(b), the two-photon joint spectra of output photons at *σ*_*m*_ are also little different in shape from those in Fig. 3(c), except for further broadening of pulse width: $2{\rm{\Gamma }}=1.23$ eV for *a* = 20 nm. Though not shown in the figure, for other *σ*_*m*_ of *a* = 10 and 15 nm, the two-photon joint spectra of the outputs are little different in shape from Fig. 4(b), except for the value of pulse width $2{\rm{\Gamma }}$. Thus, for aluminium nanospheres, further broadening of pulse width up to 1.23 eV and shorter central wavelength of 215 nm close to the deep ultraviolet region can be expected by utilising our cavity-plasmon system.

The above results of *E* for silver and aluminium nanospheres indicate that difference in material has little influence on *E*, in other words, has little influence on the mechanism of frequency-entanglement generation of our proposed method. This is because that the nonlinear effect that causes entanglement generation in our method is the absorption saturation effect of LSP. In contrast to conventional methods for generating entangled photons, such as parametric down-conversion and two-photon cascade emission, absorption saturation arises mainly from the incident power of light rather than the details about the quantum state of material. In our cavity-plasmon system, LSP can coherently and efficiently interact with incident photons through cavity modes owing to the condition of $\kappa  > g > \gamma$ so that the LSP can be saturated by one-photon absorption. Therefore, either one of the two input photons is absorbed by the LSP and the other remains unabsorbed owing to the absorption saturation effect of LSP. Consequently, photon entanglement is generated by the interference between the unabsorbed photon and the photon re-emitted from the LSP^35^.

**Figure 5:**
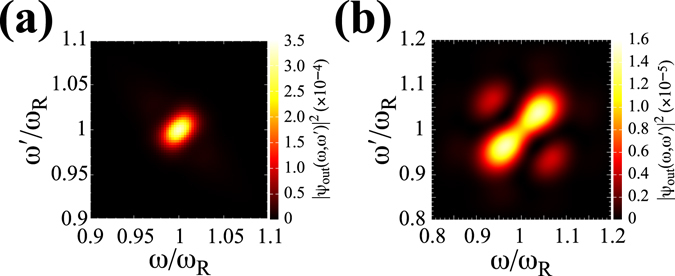
${|{\psi }_{{\rm{o}}{\rm{u}}{\rm{t}}}(\omega ,\omega \text{'})|}^{2}$ obtained from *a* = 10 nm in Fig. 3(a): (a) *σ* = 10 fs and (b) *σ* = 2.5 fs.

We finally refer to the recovery of *E* around *σ* = 10 fs observed for small nanospheres of *a *< 20 nm in Fig. 3(a). The two-photon joint spectra of output photons for *a* = 10 nm at the local minimal values of *σ* = 10 fs and at *σ* = 2.5 fs are shown in Fig. 5. In contrast to the case of *σ*_*m*_ yielding the maximum *E*, photon distribution with positive frequency correlation appears. This photon pair with positive frequency correlation is referred to as a difference-beam (DB) state, obtained by extending the conventional phase-matching condition^40^. The DB state possesses the property that two photons in a pair distributes at a distance symmetrically on the centre of a wave packet, in other words, the two photons have an inherent time delay. This property can be applied, e.g. to enhancements of up-conversion process^41^ and of two-photon process in molecules via internal conversion^42^. Though *E* is not so large, this result indicates that our proposed cavity-plasmon system can also generate another type of frequency-entangled photon pair with positive frequency entanglement, by choosing the pulse width of input photon pulse.

## Summary and Discussion

In summary, by introducing a simple quantisation method of LSP and a one-dimensional cavity-plasmon system, we have proposed a simple method for generating broadband ultraviolet frequency-entangled photons by utilising cavity quantum plasmonics. The one-dimensional cavity-plasmon system can be achieved by confining a single nanometal in a microcavity whose cavity QED parameters satisfy the condition $\kappa  > g > \gamma$. For sliver and aluminium nanometals as examples, we have shown that a frequency entangled photon pair with broadband (up to 1.23 eV), ultraviolet (215 nm) and a degree of entanglement of $E\approx 0.5$ can be generated by utilising the absorption saturation effect of LSP, which is ensured by realising the condition of $\kappa  > g > \gamma$. Under this condition, LSP can coherently and efficiently interact with incident photons through cavity modes and can be saturated by one-photon absorption. Therefore, either one of two input photons is absorbed by the LSP and the other remains unabsorbed owing to the absorption saturation effect of the LSP. Photon entanglement is formed as a consequence of the interference between the unabsorbed photon and the photon re-emitted from the LSP.

In actual experiment for the cavity-plasmon system, it might be difficult to confine a single nanometal in a microcavity, without a state-of-the-art technology in the field of cavity QED. In such a case, we can simply expand the theory by replacing a dipole representation ***p*** with a macroscopic polarisation ${\boldsymbol{P}}={\boldsymbol{p}}N/V$, where *N* is the number of nanometals and *V* is the cavity mode volume. If the interaction between nanometals in the microcavity is not so strong, the theory proposed in this study is directly applicable, though we have to slightly modify the cavity QED parameters of the cavity-plasmon system. In comparison with a similar method proposed in ref. 13, in which a nanoantenna consisting of a pair of nanometals is used, this work has a significant advantage that we do not have to manipulate nanometals, e.g. distance between the nanometals. In addition, if cluster of nanometals can be optimised, the ensemble of nanometals might exhibit superradiance phenomena, as found in quantum dots^43^. Generally, superradiance significantly shortens radiative decay time and therefore further broadening of frequency-entangled photon might be expected. On the other hand, if the interaction between nanometals is strong, Rabi splitting appears in the energy levels of LSPs and therefore the energy-level structure becomes complicated. As a consequence, the optimisation condition of the cavity-plasmon parameters obtained in this study would change significantly and further analysis would be required. This will be an issue in the future. We hope that our results will facilitate the application of entangled photons to molecular science and coherent control techniques.

## Methods

### Numerical calculation

We numerically solve equation (13) by discretising the continuous photon fields by converting from ${({\rm{\Delta }}\omega )}^{-1}\int d\omega$ and ${({\rm{\Delta }}\omega )}^{\mathrm{1/2}}\hat{a}(\omega )$ to ${\sum }_{k}$ and ${\hat{a}}_{k}$, respectively, where ${\rm{\Delta }}\omega =2\pi c/L$ is the mode spacing and *L* is the length of the calculation region. ${\rm{\Delta }}\omega$ is set to ${\rm{\Delta }}\omega =1.8$ meV. For aluminium nanospheres the plasmon parameters are set to $\hslash {\omega }_{P}=15.8$ eV and ${\varepsilon }_{\infty }\mathrm{=3.5}$, leading to $\hslash {\omega }_{R}=5.769$ eV (215 nm); for silver nanospheres the plasmon parameters are set to $\hslash {\omega }_{P}=11.585$ eV and ${\varepsilon }_{\infty }=8.926$, leading to $\hslash {\omega }_{R}=3.222$ eV (385 nm) by reference to the theoretical and experimental results^14, 18^. For a microcavity system, we assume ${\varepsilon }_{m}=2$ and a $\lambda \mathrm{/2}$ microcavity, realisable by state-of-the-art techniques in the field of cavity QED. The cavity damping rate $\kappa$ is chosen so as to satisfy $\kappa  > g$ and yield maximum *β*. For the cavity-plasmon system with silver nanometal, ${\rm{\Gamma }}=39$ meV, *γ* = 2.2 meV and *β* = 0.95 for *a* = 10 nm; ${\rm{\Gamma }}=71$ meV, *γ* = 7.3 meV and *β* = 0.91 for *a* = 15 nm; ${\rm{\Gamma }}=109$ meV, *γ* = 17 meV and *β* = 0.86 for *a* = 20 nm and; ${\rm{\Gamma }}=153$ meV, *γ* = 34 meV and *β* = 0.82 for *a* = 25 nm. For the cavity-plasmon system with aluminium nanometal, ${\rm{\Gamma }}=218$ meV, *γ* = 38 meV and *β* = 0.85 for *a* = 10 nm; ${\rm{\Gamma }}=399$ meV, *γ* = 130 meV and *β* = 0.75 for *a* = 15 nm; and ${\rm{\Gamma }}=615$ meV, *γ* = 308 meV and *β* = 0.67 for *a* = 20 nm.

### Quantum entanglement measure

When two (or many) interacting systems are in the form of an inseparable quantum state, the system is said to be entangled. We evaluate the degree of entanglement of output photons by using the entropy of entanglement. When the density operator *ρ* is in a completely pure state, the entropy of entanglement uniquely determines the degree of entanglement of *ρ*. There are some quantum entanglement measures, namely entanglement of formation^38^ and relative entropy of entanglement^44^. Generally, relative entropy of entanglement is used for mixed states not considered in this study, and therefore we adopt the entanglement of formation. Entanglement of formation *E* is defined as $E=-{\rm{T}}{\rm{r}}[\rho \text{'}{\mathrm{log}}_{d}\rho \text{'}]\,{\rm{w}}{\rm{i}}{\rm{t}}{\rm{h}}\,\rho \text{'}={\rm{T}}{\rm{r}}\text{'}[\rho ]$, where $\rho =|\psi \rangle \langle \psi {|}_{{\rm{out}}}$ is the density operator of two output photons; $\rho \text{'}$ indicates the density operator partially-traced for one photon and *d* is the dimension of *ρ*. *E* = 1 indicates that output photons are fully entangled, whereas *E* = 0 implies there is no quantum entanglement in the output photons. In the actual calculation, we evaluate *E* for the output photons far from the cavity-plasmon system.
